# Characterizing Infiltrating Macrophages in Intracranial Aneurysm with IA Animal Models and Spatial Transcriptomics

**DOI:** 10.1007/s12975-026-01437-6

**Published:** 2026-04-29

**Authors:** Hosu Kim, Tomohiro Aoki, Masahiko Itani, Isao Ono, Yong-Hee Kim, Chung-Gyu Park

**Affiliations:** 1 Translational Medicine Major, Department of Medicine, Seoul National University College of Medicine, Seoul, Republic of Korea; 2 Department of Pharmacology, the Jikei University School of Medicine, Tokyo, Japan; 3 Department of Neurosurgery, Kyoto University Graduate School of Medicine, Kyoto, Japan; 4 Department of Neurosurgery, Hikone Municipal Hospital, Shiga, Japan; 5 Transplantation Research Institute, Medical Research Center, Seoul National University College of Medicine, Seoul, Republic of Korea; 6 Department of Biomedical Sciences, Seoul National University College of Medicine, Seoul, Republic of Korea; 7 Department of Microbiology and Immunology, Seoul National University College of Medicine, Seoul, Republic of Korea; 8 Institute of Endemic Diseases, Seoul National University College of Medicine, Seoul, Republic of Korea; 9 Cancer Research Institute, Seoul National University College of Medicine, Seoul, Republic of Korea; 10 BK21 Plus Biomedical Science Project, Seoul National University College of Medicine, Seoul, Republic of Korea

**Keywords:** Intracranial aneurysm, Macrophages, Animal models, Spatial transcriptomics

## Abstract

Intracranial aneurysm (IA) prevalence in the general population can be as high as 3%. Most IAs are asymptomatic and diagnosed incidentally. However, IAs can cause subarachnoid hemorrhage (SAH), which is responsible for high mortality and morbidity. No effective medical therapy is currently available to control the IA and reduce the SAH risk. Understanding IA pathophysiology to identify potential targets is, therefore, an essential clinical objective. IAs typically form at arterial bifurcations and are characterized by macrophage infiltration. Macrophages are innate immune cells that present antigens to adaptive immune cells and promote inflammation. The potential role of macrophages in IA was demonstrated when inhibiting the macrophage recruitment contained aneurysm formation or growth. Recent advances in spatial transcriptomics enable the study of infiltrating macrophages within the aneurysmal lesion and the identification of pathogenic changes that were previously challenging to probe. While sample availability may limit the use of spatial transcriptomics, judicious use of IA animal models will enable translational research to address unmet clinical needs. With spatial insights and refined animal models, researchers can identify key characteristics of infiltrating macrophages, including their ontogeny, transcriptomic profiles, and interactions with other cell subsets. This review article summarizes the current understanding of infiltrating macrophages in IAs and introduces available animal models of IA and spatial transcriptomics approaches for studying macrophage infiltration.

## Introduction

Intracranial aneurysms (IAs) are common in the general population, with a reported prevalence of up to 3% [1, 2]. Ruptured IAs lead to subarachnoid hemorrhages (SAH), associated with high mortality and morbidity [3–5]. Currently, no approved medical therapy exists to prevent the development or progression of IA [6]. Surgical or endovascular procedures carry a non-negligible risk, and there is a definite unmet clinical need for effective medical therapy [7, 8].

Understanding the immunological contributions to disease pathogenesis can accelerate the development of medical therapies. For example, improved understanding of the PD-1/PD-L1/CTLA-4 axes has led to the development of effective anticancer immunotherapies [9–12]. The initial trigger and the factors that promote growth and rupture require further investigation. However, non-physiological hemodynamic forces, low wall shear stress, and disrupted laminar flow are thought to contribute to the pathophysiology of IA [2, 13, 14]. The later stage involves a weakened arterial wall, enlarged by hemodynamic forces, with immune cell infiltration. The role of immune cell infiltration remains poorly defined, and understanding the mechanisms underlying these cells could provide valuable insights for the development of medical therapies [2].

Macrophages account for the bulk of immune cell infiltration [15–18]. Inhibiting macrophage infiltration also reduced IA growth and rupture in animal studies [19–21]. However, the causal relationship between macrophage infiltration and IA remains unclear and warrants further investigation. The immune system comprises heterogeneous cell populations, and probing a specific cell subset for its contribution to pathology can therefore be challenging. Although there is no single perfect toolkit, spatial transcriptomics projects RNA expression patterns onto tissue architectures, enabling the discovery of previously unrecognized structures and an in-depth understanding of immune cell spatial distribution and activity profiles [22, 23]. For instance, Lai et al. used Stereo-seq, in addition to single-cell transcriptomics, to identify previously unrecognized tertiary lymphoid organs in carotid plaques. They demonstrated robust B-cell activity in these structures in patients undergoing carotid endarterectomy [24].

In IAs, infiltrating macrophage transcriptomics can be analyzed spatially, revealing their presence in the tunica intima, media, and adventitia, as well as adjacent brain or extracranial lymphoid structures. Unlike single-cell transcriptomics, spatial transcriptomics can determine whether infiltrating macrophages in the tunica intima and adventitia share similar transcriptomic profiles, providing insights difficult to obtain with single-cell transcriptomics alone [23, 25–27]. A key obstacle to studying infiltrating macrophages in IAs using spatial transcriptomics is the limited availability of high-quality human samples. Collaboration will be required to collect enough samples. Established animal models will mitigate some of the difficulties. As with other diseases, the individual IA clinical courses will depend on aneurysm size, location, morphology, risk factors, and other demographic information such as age or sex [28, 29]. Accounting for disease heterogeneity will be important when studying infiltrating macrophages using spatial transcriptomics. For example, IAs at arterial bifurcations are exposed to distinct hemodynamic stresses, which are likely to alter transcriptomic patterns via mechanotransduction [30]. Finally, female sex is a risk factor, and aneurysms frequently rupture in postmenopausal women; the sexual dimorphism, the sex hormones, and their potential influences on the IA pathology warrant a close investigation [2, 29, 31].

### The Anatomy of IA

**Fig. 1:**
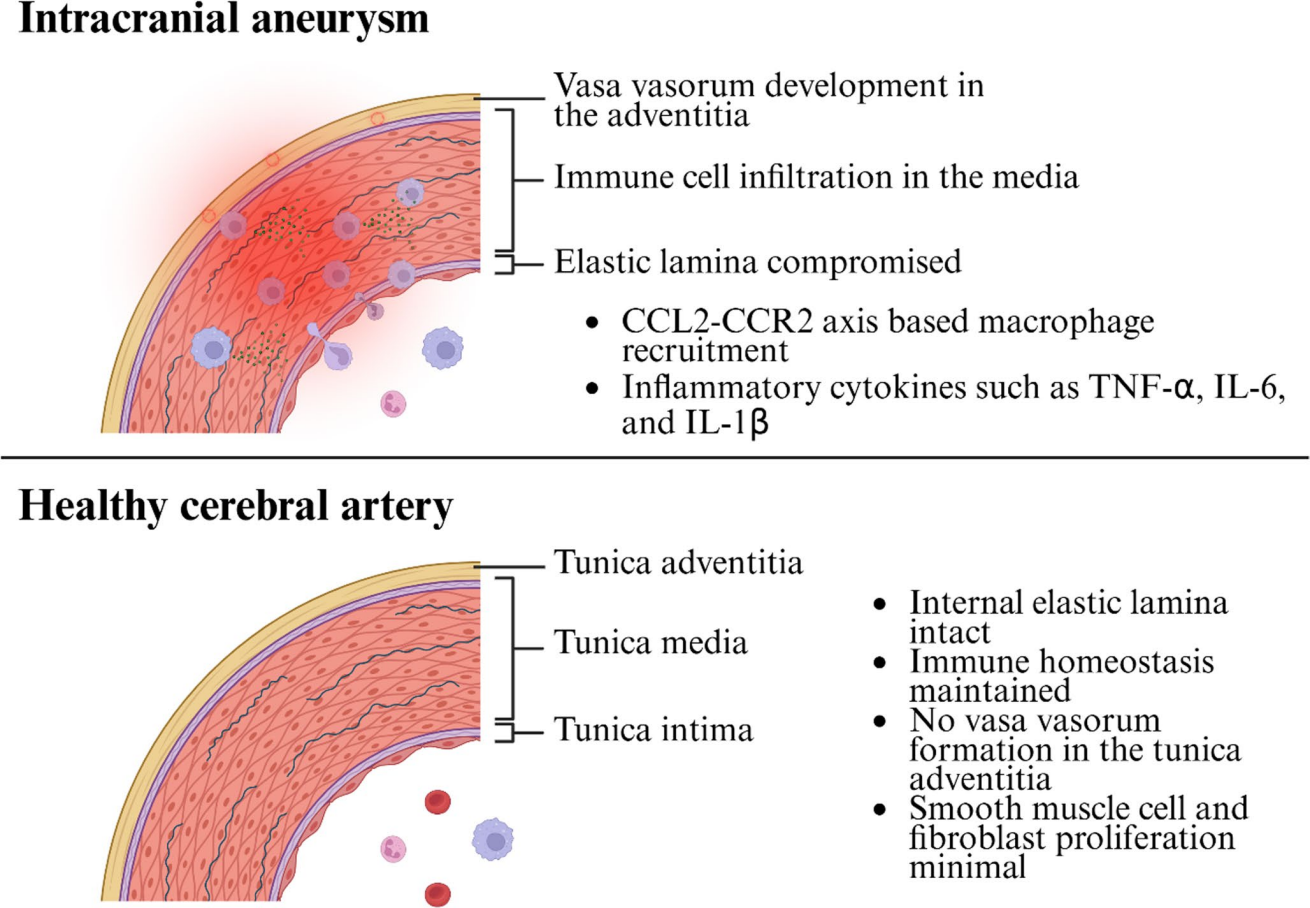
The illustrations depicting the healthy cerebral artery (bottom) and IA (top) are shown. In IA, the elastic lamina is compromised, and macrophage infiltration is observed. Vasa vasorum also form in the tunica adventitia. Monocytes, macrophages, and neutrophils are depicted infiltrating IAs in the top illustration. IA, intracranial aneurysm; CCL2, C-C motif chemokine ligand 2; CCR2, C-C motif chemokine receptor 2; TNF-α, tumor necrosis factor-alpha; IL-6, interleukin-6; IL-1β, interleukin-1 beta

IAs can be classified into either saccular or non-saccular (fusiform, dissecting, etc.) aneurysms depending on the morphology. Saccular and non-saccular aneurysms differ in growth and rupture risk [32]. The review will focus only on the saccular IAs. Most IAs are formed along the Circle of Willis (CoW) (Fig. 1). For example, the anterior communicating artery IAs account for approximately 18% of the total IA prevalence. The cerebral arteries that consist of the CoW are the anterior cerebral artery (ACA), the anterior communicating artery (ACom), the middle cerebral artery (MCA), the internal carotid artery (ICA), the posterior cerebral artery (PCA), the posterior communicating artery (PCom), and the tip of the basilar artery (BA). ACA, ACom, and ICA make up the anterior cerebral circulation. The posterior circulation comprises the PCA, BA, and vertebral arteries (VA). While most IAs are observed in the anterior circulation, IAs can form anywhere inside the cranium [33].

Arteries within the cranium are structurally distinct from peripheral arteries despite similarities (Fig. 1). Cerebral arteries have thinner tunica media and lack an external elastic lamina, rendering them more susceptible to hemodynamic stress. Furthermore, cerebral arteries typically lack vasa vasorum and develop them in pathologic conditions, such as IAs [34, 35]. In IAs, macrophages extensively infiltrate the cerebral arteries, with cells present in the tunica intima, media, and adventitia [15–17]. While the exact origin of infiltrating macrophages remains unclear, they may be partially recruited through the vasa vasorum and cross the endothelial barrier [36, 37].

In addition to cerebral arterial structure and immune cell infiltration, arterial bifurcations also contribute to IA pathogenesis. Pulsatile hemodynamic stress can batter the endothelium at arterial bifurcations [13, 30]. The fact that mechanical stress can trigger immune responses is well known [38]. For instance, macrophages cultured under high pressure release more inflammatory cytokines than those cultured under low pressure [38]. Given the time required for IAs to progress, aneurysm formation likely involves chronic inflammation; the hemodynamics-mediated immune response is a plausible source of this inflammation.

There are several well-established risk factors associated with IA formation, such as smoking, older age (> 50 years), female sex, genetic diseases like autosomal dominant polycystic kidney disease, and hypertension [2, 29, 32]. Female sex acts as a protective factor against abdominal aortic aneurysm but as a risk factor for IAs. Although type 2 diabetes is a risk factor for atherosclerosis and other vascular diseases, it might be a protective factor in IA, potentially suggesting heterogeneous mechanisms between atherosclerosis and IA [39]. How each clinical risk factor translates into higher IA incidence and growth over time remains to be established [2].

### Infiltrating Macrophages in IAs

Most infiltrating macrophages derive from circulating monocytes. When an inflammation occurs, monocytes are mobilized and adhere to the vascular endothelium. Afterward, monocytes infiltrate the vessel and differentiate into macrophages. Infiltrating macrophages can release inflammatory cytokines, including TNF-α, IL-6, and IL-1β [2]. In addition to releasing inflammatory cytokines, macrophages can contribute to vessel wall degradation by secreting proteases, including matrix metalloproteinases (MMPs) [40]. In the IA context, MMP-2 and MMP-9 are considered salient in the disease development [40–42]. Pathologic investigations have revealed substantial complement deposition in IA, and the complement system is another pathway through which infiltrating macrophages could contribute to IA pathogenesis [43–45]. As antigen-presenting cells (APCs), differentiated macrophages can also take up antigens and present them to adaptive immune cells, such as T cells, in secondary lymphoid organs [46]. Activated T cells, in turn, can stimulate macrophages, increasing their longevity and inflammatory potential.

When monocytes are recruited during inflammation and differentiate into monocyte-derived macrophages (MDMs), the cells can constitute a significant proportion of the tissue-resident macrophage population [47]. For example, tissue-resident macrophages in the adult heart primarily derive from MDMs. On the other hand, there are organs where the MDM recruitment rarely occurs. Tissue-resident macrophages in closed organs, such as the brain, derive from embryonic and fetal liver macrophages and persist through self-renewal [48]. Macrophage-derived microglia, as well as border-associated macrophages (BAMs), populate the brain parenchyma, meninges, and choroid plexus through self-expansion [49]. Given the IA’s proximity to the brain, BAMs may be present at an aneurysmal lesion when the IA gets sufficiently enlarged. On the other hand, due to anatomical constraints, such as the cerebrospinal space, recruitment of BAMs to the IA site may be limited, and infiltrating macrophages may predominantly be MDMs. Investigating ontogenic similarities between infiltrating macrophages and BAMs will help determine whether infiltration results from the recruitment of BAMs or MDMs, providing additional insights for future therapeutic development.

**Fig. 2:**
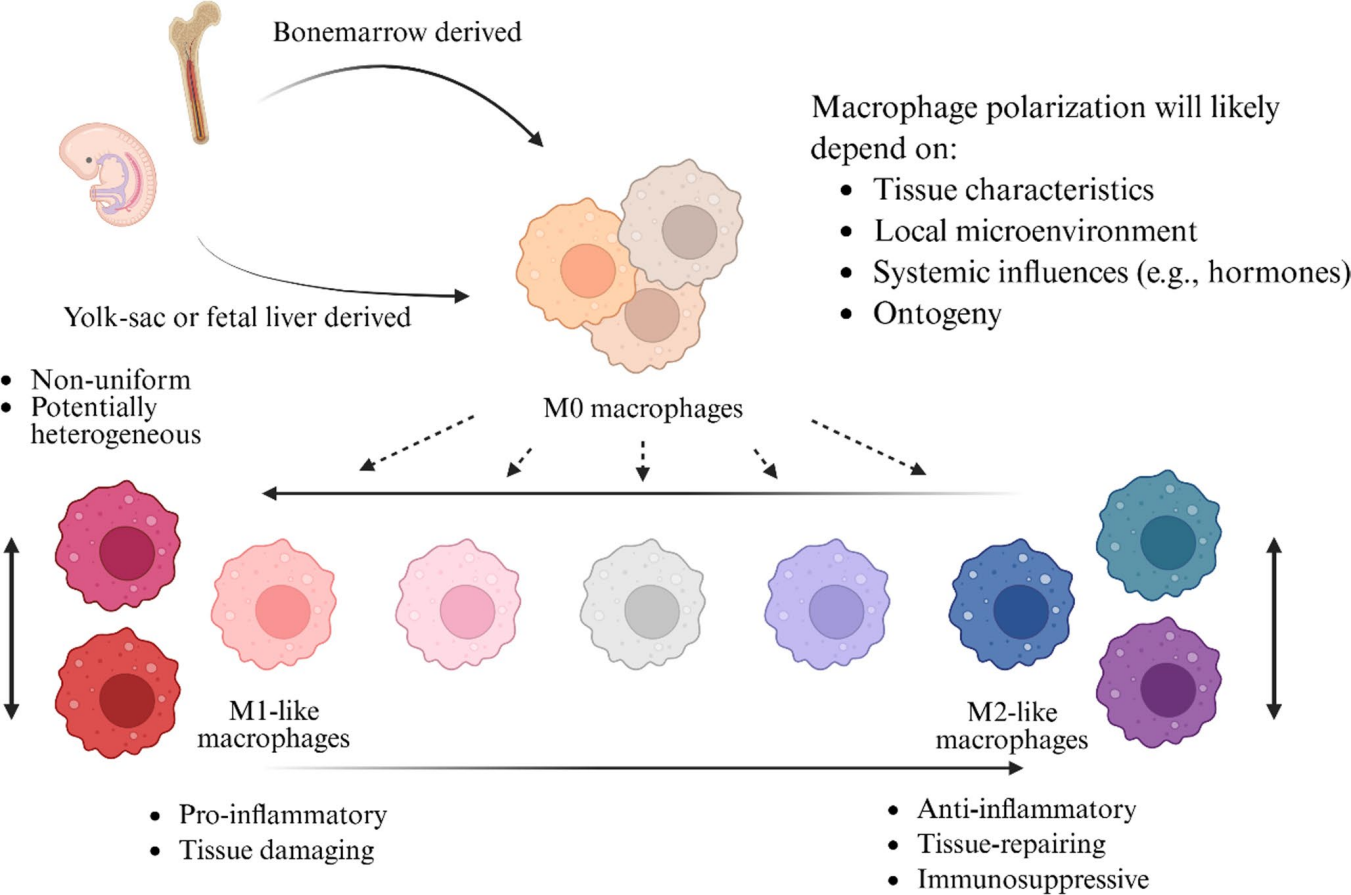
The macrophage states, such as classical M1-like and M2-like, are shown. The origins of BAMs (yolk-sac or fetal liver) and MDMs (bone marrow) are also illustrated. M1-like and M2-like states will depend on factors such as tissue characteristics, local microenvironment, systemic influences, and ontogeny. While M1-like and M2-like states can be used, caution is warranted, as these terms may indicate heterogeneous macrophage states across contexts. BAM, brain-associated macrophage; MDM, monocyte-derived macrophage

Macrophages can be classically categorized into M1 and M2 macrophages. However, it is worth noting that the M1/M2 dichotomy is now understood to be overly simplistic. Macrophage states, such as M1 or M2, are better described as specific states along a continuum (Fig. 2). Therefore, while we use terms such as classical M1-like and M2-like macrophages in this review, we caution readers about the limitations of these terms; henceforth, we simplify them to M1- and M2-like. Furthermore, in the field of IA research, characterization of macrophage states has been limited to M1- and M2-like states. In this aspect, spatial transcriptomics could provide novel insights into macrophage states in IA [50–54]. M1-like macrophages are pro-inflammatory, whereas M2-like macrophages suppress inflammation and promote tissue repair [55]. Although reports are inconsistent, it has been reported that M2-like macrophages are more common than M1-like macrophages at ruptured aneurysms, whereas the opposite is observed in unruptured IAs [15–17] (Table 1). Although the relationship between macrophage phenotype and aneurysmal rupture remains unclear, phenotypic changes in macrophages may be closely associated with rupture [17]. However, the macrophage-phenotype efforts described in Table 1 had technical limitations (e.g., reliance on limited markers such as HLA-DR for M1-like and anti-CD163 for M2-like). Once again, spatial transcriptomics could more effectively identify specific macrophage states associated with aneurysmal rupture through more comprehensive spatial transcriptomic signatures [23, 50, 52, 54].

**Table 1:** Investigations that examined macrophage phenotypes in human intracranial aneurysms and animal models

Investigation	Sample type (Unruptured vs. ruptured)	Investigation method	Macrophage phenotype (M1-like vs. M2-like)	Sample size/ sex ratio	Limitations and considerations	Additional comments
Human sample investigations						
Hasan et al., 2012 [15]	5 unruptured and 5 ruptured samples	Immunostaining with monoclonal antibodies (M1-like: anti-HLA DR; M2-like: anti-CD 163)	Equal proportions in unruptured samples; M1-like dominance in ruptured samples	*n* = 10; 5 female samples (2 ruptured); 5 male samples (3 ruptured)	A relatively small sample size	Mast cells also found to be upregulated in ruptured aneurysms. All layers (intima, media, and adventitia) analyzed.
Yamashiro et al., 2019 [16]	1 unruptured and 7 ruptured (2 with concurrent subdural hematoma)	Immunostaining with monoclonal antibodies (M1-like: anti-HLA DR; M2-like: anti-CD 163/204)	No statistical difference was found; M1 and M2-like were both present	*n* = 8; 7 female samples (6 ruptured); 1 male sample (ruptured)	A relatively small sample size. Mostly ruptured female samples	For ruptured cases: M1-like > M2-like (2 cases), M1-like < M2-like (2 cases), M1-like = M2-like (3 cases). All layers analyzed.
Stratilová et al., 2023 [17]	28 unruptured and 13 ruptured samples	Immunostaining with monoclonal antibodies (M1-like: anti-HLA DR; M2-like: anti-CD 163)	M2-like dominance in ruptured samples; M1-like dominance in unruptured samples	*n* = 41; 29 female samples (10 ruptured); 12 male samples (3 ruptured)	A smaller proportion of ruptured samples	unruptured IAs morphologically similar to ruptured IAs had intraluminal thrombi. All layers analyzed.
Animal model investigations						
Investigation	Species	Investigation method	Macrophage phenotype (M1-like vs. M2-like)	Sample size/ sex ratio	Limitations and considerations	Additional comments
Nowicki et al., 2017 [20]	Mouse	Immunostaining with monoclonal antibodies (M1-like: F4/80, iNOS; M2-like: F4/80, Arg I)	M1/M2-like ratio 0.56 at 3 days to 1.75 at 2 weeks	Female C57BL/6 mice (*n* = 5, 3 days; *n* = 6, one week; *n* = 25, two weeks)	The study was designed to test the anti-CXCL-1 antibody. The control mice were injected with IgG control	Only the control mice information described
Khashim et al., 2020 [122]	Rabbit	Immunostaining with monoclonal antibodies (M1-like: CD80; M2-like: CD206)	M1-like number highest at 1 month time point. M2-like number highest at 6-month time point	New Zealand white female rabbits (*n* = 6, 1 month posttreatment; *n* = 5, 3 months; *n* = 6, 6 months)	Rabbits were treated with endovascular coiling treatment after aneurysm induction	M1 and M2-like macrophages not directly compared. Histologic score graded

**Fig. 3:**
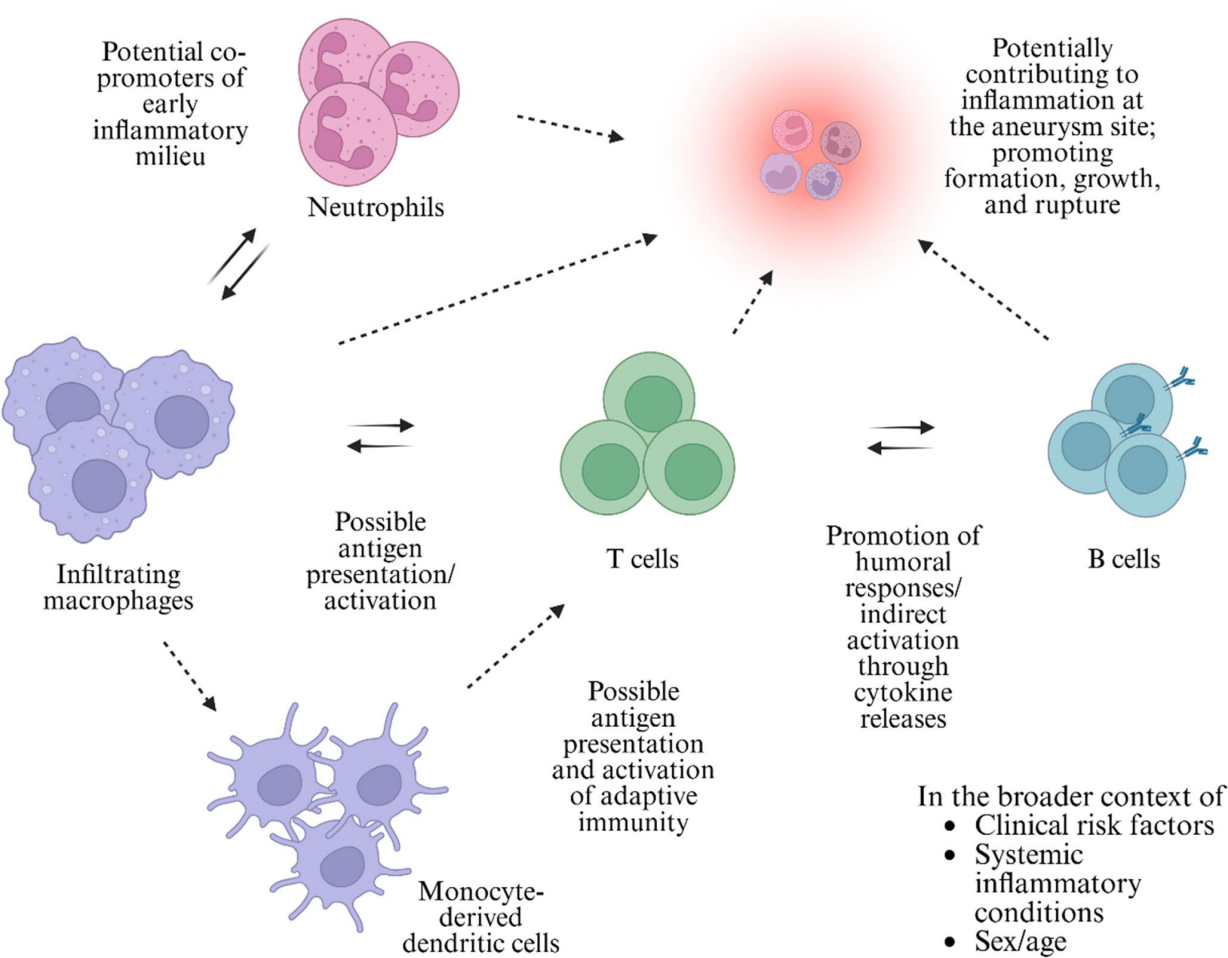
Infiltrating macrophages and their potential interactions with other immune cell groups are illustrated. Interactions less grounded by previous research are indicated in dashed lines. While commonly accepted interactions are indicated by solid lines, the IA context could influence immune interactions. The broader context, including clinical risk factors, systemic inflammation, sex, and age, should be considered when evaluating immune cell interactions

Infiltrating macrophages could contribute to IA pathogenesis by promoting the generation of monocyte-derived dendritic cells (Mo-DCs) [56]. Given the chronicity of IA pathogenesis, it is possible that the pathology involves both innate and adaptive immune responses, such as a specific CD4 + T helper cell axis (Fig. 3). Given DCs’ capacity as professional APCs, Mo-DCs could serve as the link between innate and adaptive immunity in the context of IA. Although discovered in the context of multiple sclerosis, the blood-brain barrier (BBB) has been reported to facilitate the formation of a microenvironment conducive to Mo-DC, specifically for monocyte differentiation into Th17-polarizing DCs [57]. A similar effort to characterize Mo-DC in the IA context could yield valuable insights.

While tissue-resident macrophages have long been presumed to be sessile, recent reports suggest that some are mobile and rapidly migrate to sites of injury [58, 59]. The BAMs and microglia populations derive from yolk-sac progenitor cells and are ontogenically distinct from bone marrow-derived MDMs. In neurodegenerative and neuroinflammatory disorders, a greater percentage of BAMs and microglia are replaced by MDMs [60]. However, MDM-origin BAMs and microglia are transcriptomically distinct from their original populations and lack a bona fide microglial marker, such as SALL1 [49]. Further research is warranted to determine whether MDM-origin BAMs and microglia are byproducts of neuroinflammation or directly contribute to neurodegeneration and neuroinflammation [61].

Hypotheses such as the idea that infiltrating macrophages can bolster the adaptive immune response by directly presenting antigens or facilitating Mo-DC differentiation could be beneficial in understanding IA growth. The properties of the BBB in the IA context warrant further research. Finally, the fact that infiltrating macrophages or monocytes replace incumbent BAMs and microglia, and that they affect the central nervous system (CNS) immune system, raises further questions about their potential role in IAs. Nevertheless, it might not be sufficient to account for the female predilection and anatomical preferences for bifurcations in IA formation and development. Two additional factors could be pertinent to understanding the role of infiltrating macrophages in IA pathology: sexual dimorphism and hemodynamics-mediated immune activation [2, 38].

### Sexual Dimorphism

Like every other cell, infiltrating macrophages exhibit sexual dimorphism [62]. Taking into account that female sex is a proven risk factor, it is essential to investigate the influence of sexual dimorphism on the infiltrating macrophages and their connection to the IA pathology [29, 31]. In fact, macrophages have been widely implicated in various female reproductive disorders [63]. Moreover, the sexual dimorphism in the immune system is widely and consistently reported [62, 64, 65].

Given the heterogeneity of the immune system, infiltrating macrophages in IAs may exhibit distinct sexual dimorphism [47, 62]. Interpretation requires caution when studying sexual dimorphism at other anatomical sites and in other cell groups, and insights can still be obtained. For instance, Chi et al. elucidated the mechanism underlying skin immune enrichment in females by showing that unstimulated skin immunity differs between male and female mice [65]. Testosterone was found to decrease the type 2 innate lymphoid cell (ILC) population via the androgen receptor (AR), which is abundantly expressed in these cells. By contrast, the abundance of type 2 ILCs in females expanded DC populations by secreting greater amounts of granulocyte-macrophage colony-stimulating factor (GM-CSF). Nonetheless, the relevance of ILCs in the IA and CNS contexts warrants further investigation and characterization [66, 67].

It should also be noted that aneurysms rupture more frequently in post-menopausal women, implying that estrogen depletion leads to exacerbation of the aneurysm [2]. Estradiol, an active form of estrogen, has been reported to promote M2 polarization in vitro and to increase IL-10 secretion, thereby attenuating inflammation [62]. Estradiol has also been reported to modulate microglial activity in the brain [68]. Animal models are well-suited to investigating IA sexual dimorphism with maneuvers such as ovariectomy or hormone replacement therapy [69]. Well-established models will mitigate difficulties for procuring patient samples and fill gaps in translational research [70].

### Mechanotransduction

Physical stimuli have long been recognized as sources of immune reactions—for example, dermatographism appears after mechanical scratching. Immune cells are equipped with sensors, including TRPV4 and Piezo1, and can respond differently to identical stimuli under different pressures [38]. For example, APCs activate T cells more readily when the lymph node is swollen [38]. Similarly, physical stimulation of tissues or organs can activate innate immune cells and induce the release of inflammatory cytokines. The influence could be either direct or indirect. For example, Piezo1 + enteric neurons can indirectly modulate the intestinal immune response in response to mechanical forces [71]. Moreover, external pressure can affect interactions between endothelial cells and circulating immune cells, including monocytes and neutrophils. As a result, more immune cells can extravasate or infiltrate blood vessels, where endothelial cells are subject to external pressure. Recent investigations of endothelial cells suggest that cellular and mitochondrial membranes may change in response to shear stress induced by laminar-flow disruption [72]. Given that endothelial cells can act as APCs, hemodynamics-mediated membrane changes could affect their APC function by modulating surface MHC expression in humans [73].

Hemodynamics at arterial bifurcations expose infiltrating macrophages and their neighboring cells to turbulence. In fact, most IAs develop at arterial bifurcations, where laminar flow can be disrupted, and endothelial cells are exposed to shear stress [30]. While it is unclear whether hemodynamics modulate IA pathogenesis directly or indirectly, it is desirable to account for temporal effects, as cells may be shaped differently at different time points. For example, turbulence-mediated mechanotransduction may play different roles depending on temporal influences. It might be challenging to directly investigate the contribution of the mechanotransduction to the IA pathogenesis. However, the broader research field studying the biology of age-associated vascular stiffening (over the lifespan) or short-term temporal effects (e.g., circadian rhythms) might provide indirect clues regarding the contribution of mechanobiology to early versus late-stage IA [74–77]. Understanding the mechanisms by which hemodynamics translate into IA could provide novel insights into the development of therapeutics and the prediction of high-risk IAs that are difficult to detect among low-risk IAs [6, 78, 79].

### Infiltrating Macrophage Interaction with Immune Cells and Microbiome

Macrophages migrate to sites of inflammation by differentiating from monocytes. Furthermore, resident macrophages adapted to their environments normally reside in tissues and organs. The best-known examples are microglia and BAMs in the brain. Having been present in tissues since birth or recruited by inflammation, macrophages interact with other immune cells also present at the site. The macrophage’s interaction with other immune cells will thus depend on location, environmental characteristics, and systemic conditions, such as infection [47–49].

In the IA context, infiltrating macrophages will inevitably be in proximity to neutrophils, the cells that mobilize during early inflammation. Neutrophils arrive at the inflammatory site within a few hours and are known to precede macrophages [58, 80]. On the other hand, a recent report suggests that macrophages precede neutrophils at a sterile peritoneal injury [59]. Regardless of recruitment order, macrophage interactions with neutrophils will likely influence macrophage activity, which is highly plastic [81, 82]. In addition to neutrophils, macrophages or monocytes must interact with vascular endothelial cells to successfully infiltrate the intracranial artery. While the vessel lumen will consist primarily of endothelial cells, myofibroblasts or fibroblasts in general may be present within the vessel architecture, particularly in the adventitia, and may contribute to disease progression [83].

T and B lymphocytes will also be pertinent in IA pathology. Given the chronic nature of IA pathogenesis, the adaptive immune system is likely involved in IA growth and rupture. Macrophages and Mo-DCs can migrate to secondary lymphoid organs, where they function as APCs and activate T lymphocytes specific for self- or foreign antigens present at the IA lesion [56]. T lymphocytes can then migrate to the IA site or activate B lymphocytes in the germinal centers [84]. Alternatively, an adaptive immune response triggered by a systemic cause could indirectly contribute to IA or SAH, as in the case of infection. Although viral infections were not clearly associated with IA rupture, it remains possible that adaptive immune activation via cross-reactivity contributes to disease development [85]. In the recent COVID-19 pandemic, SAH cases were reported in one male and five female patients after the COVID-19 infections [86]. Indeed, severe COVID-19 cases can result in a cytokine storm and the indiscriminate activation of immune cells [87]. However, the possible confounding effects of ventilator-associated blood pressure fluctuations and other systemic comorbidities warrant further evaluation [86, 88].

Microbiome dysregulation can also lead to immune-mediated inflammation. Hallikainen et al. reported a higher prevalence of gingivitis and periodontitis among IA patients than among controls, suggesting dysregulation of the oral microbiota in IA [89, 90]. The study also found significantly higher serum IgA titers and lower IgG titers against *Porphyromonas gingivalis* and *Aggregatibacter actinomycetemcomitans* in patients with IA [89, 90]. Moreover, a gut microbiome study of patients with unruptured and ruptured IA found that tryptophan metabolite data could distinguish ruptured from unruptured IA with high accuracy (AUC = 0.97) [91]. Given that the constitution of CNS humoral immunity depends on the gut microbiome, further research is warranted to elucidate the role of microbiota in IA pathogenesis [92].

### IA Animal Models

Since Hashimoto introduced the classical IA rat model, many refinements and variations have been developed for IA translational research [70, 93–95]. While large animals, such as pigs and primates, can be used in spatial transcriptomics research, handling, associated costs, and ethical considerations can be prohibitive [93]. The review article will focus on the Hashimoto rodent models (named after Nobuo Hashimoto and Tomoki Hashimoto) and briefly discuss the Hassler rabbit model (Table 2). Most of the development experience has been gained using two Hashimoto rat and mouse models [93–95]. The aneurysm induction in rat models involves common carotid artery (CCA) ligation to alter the blood flow pattern in CoW, a lysyl oxidase inhibitor such as beta-aminopropionitrile (BAPN) to perturb collagen and elastin fiber cross-linking, a high-salt diet (usually with concurrent renal artery ligation), and deoxycorticosterone acetate (DOCA) to increase blood volume and induce hypertension. The original IA rat model reported a 37% aneurysm induction rate, requiring 11–21 weeks [93]. Subsequent improvements to the model led to higher induction rates and shorter times to aneurysm development. The rat model closely reproduces human pathology, is reliable, widely used, and has been reported in the literature [93, 94].

**Table 2:** A list of intracranial aneurysm animal models that can be deployed for spatial transcriptomics investigations

Model	Species	Extracranial or intracranial	IA induction method	Induction rate	Aneurysm development time	Testable pathologic elements/ Strengths
Hashimoto model (Nobuo) [93]	Rat, mouse, primates (Macaca fascicularis)	Both	Carotid ligation, lysyl oxidase inhibitor, DOCA-induced hypertension, high-salt diet, etc.	37% in the original study. Variations report higher rates	11–21 weeks after lysyl oxidase inhibitor injection. Variations report shorter times.	Hemodynamic stress [13], hypertension, vessel wall weakening, estrogen deficiency [69], etc.
Hashimoto model (Tomoki) [93]	Mouse, rat	Both	Elastase injection, angiotensin II, carotid ligation, high-salt diet, etc.	60–80%. Variations reported	2–3 weeks after induction	Similar to Nobuo Hashimoto’s model, genetically modifiable
Hassler Model [93]	Rabbit	Intracranial	Bilateral carotid ligation	100% reported in bilateral ligation	12 weeks after induction	Hemodynamic stress

The Hashimoto mouse model involves induction steps similar to those in the rat model, such as CCA ligation, a high-salt diet (sometimes with concurrent renal artery ligation), and angiotensin II administration. The mouse model, however, utilizes elastase injection in the cerebrospinal space to weaken the vessels in CoW [93]. The original model reported a high induction rate ranging from 60 to 80%. The mouse model also has a short induction period, requiring only two to three weeks for full aneurysm induction. Additional advantages include the wide availability of genetic modifications: incorporating specific genetic modifications will take less time. However, mouse models yield smaller sample sizes and may limit their utility in investigating certain questions using spatial transcriptomics [96]. For instance, the diameter of a typical aneurysm sample is approximately 20 μm in murine models and 100 μm in rat models [96]. Given the costs associated with spatial transcriptomics, it is advisable to produce a tissue microarray to investigate multiple samples simultaneously.

One important limitation of the described rodent models is that they use BAPN or elastase to weaken blood vessels for aneurysm induction. In this regard, the models depart from the more chronic, gradual pathogenesis of IA and might not completely recapitulate human IA pathophysiology; consequently, the immune environment. However, these applications closely emulate human pathology: degenerative changes in the extracellular matrix, disruption of the internal elastic lamina, and collagen fiber degradation are robustly observed. Moreover, such applications significantly facilitate the induction and growth of aneurysms in IA animals, thereby shortening the observation period and elevating the reproducibility [2, 70, 93]. Finally, both rat and mouse models report higher rupture rates with ovariectomy and lower rates after hormonal replacement, replicating the elevated rupture rate in women after the 5th decade [69, 93]. While the exact mechanism remains to be investigated, the judicious combination of IA rat and mouse models provides an opportunity to examine in depth the effect of low estrogen on IA rupture. Nevertheless, the immune system and genetics differ between humans and the models, consequently requiring caution in interpretation depending on the specific context [97–104].

Unlike rodent models, the Hassler rabbit model requires no procedure beyond bilateral carotid ligation and reports a 100% aneurysm induction rate. The model will therefore help investigate the effects of hemodynamic stress on IA pathology. The vessel caliber and aneurysm size are also larger, which may facilitate the investigation of novel medical procedures or equipment. However, its use has been more limited than in the above rat or mouse models [93]. In summary, the Hashimoto models for rat and mouse IA provide a large pool of variants for diverse translational research. The Hassler model may serve as a platform for clarifying the effects of aberrant hemodynamics and testing the latest medical procedures or equipment.

## Spatial Transcriptomic Modalities

### GeoMx Digital Spatial Profiler

Bruker GeoMx Digital Spatial Profiler (DSP) generates spatial transcriptomics using next-generation sequencing (NGS) (Table 3) [105]. Researchers can use either fresh-frozen (FF) tissues or formalin-fixed, paraffin-embedded tissues (FFPE). When using FFPE slides, researchers perform deparaffinization, antigen retrieval, and protease digestion to prepare the slides. Subsequently, RNA probes are hybridized, and immunofluorescent markers are incubated. Afterward, the slides are processed to extract RNA. Up to 4 immunofluorescent markers can be used to visualize tissue architecture. For example, researchers can use alpha-smooth muscle actin to stain smooth muscle cells and visualize tissue architecture. Regions of interest (ROIs) are then delineated to selectively extract transcriptomic data from the ROIs. For IA samples, ROIs can be drawn to encompass the rupture site or the entire aneurysmal lesion. While the shape and area of each ROI may vary depending on the research aim, it is recommended that each ROI contain at least 100 cells to ensure adequate sequencing depth.

**Table 3:** Different spatial transcriptomic methods

Method	Sequencing or image-based	Key technology	Resolution	Supported panels	Species	Sample compatibility	Sample size
GeoMx [105]	Sequencing based	Area-specific photocleavage	50 μm	Whole transcriptome atlas, mouse transcriptome atlas	Human, mouse^a^	FF and FFPE	14.6 mm x 36.2 mm
CosMx [106]	Image-based	Cyclic in situ hybridization	Subcellular	Whole transcriptome, 1 K, and 6 K.	Human, mouse	FF and FFPE	20 mm x 15 mm
Visium v2 [107, 109]	Sequencing based	Gridded barcodes	100 μm; 55 μm spot	v2 WT, v 1 3’ Gene Expression	Human, mouse; agnostic	Fixed frozen, FF, and FFPE	6.5 mm x 6.5 mm, 11 mm x 11 mm (larger)
Visium HD [109]	Sequencing based	Gridded barcodes	2 μm; 8 μm (bin)	HD WT, HD 3’ Gene Expression	Human, mouse; agnostic	Fixed frozen, FF, and FFPE	6.5 mm x 6.5 mm (two areas per slide)
Xenium [110, 111]	Image based	Padlock probes; rolling-circle amplification	Subcellular	< 500 and 5 K panels	Human, mouse; customization available	FF and FFPE	Optimal 10.45 mm x 22.45 mm (Maximum 12 mm x 24 mm)
Stereo-seq v1.3 [22]	Sequencing based	DNA nanoballs	0.5 μm. 0.22 μm spot	Whole transcriptome panel	Species agnostic	FF	0.5 cm x 0.5 cm, 1 cm x 1 cm (Up to 13 cm x 13 cm)
Stereo-seq v2 [112]	Sequencing based	DNA nanoballs	0.5 μm	Whole transcriptome panel	Species agnostic	FF and FFPE	1 cm x 1 cm

^a^Rat investigation reported [123]

### CosMx Spatial Molecular Imager

Unlike GeoMx, the CosMx Spatial Molecular Imager employs an image-based approach to acquire data at subcellular resolution and can visualize transcripts within cells. CosMx accommodates FF or FFPE specimens, and the slide preparation protocol is highly analogous to that of GeoMx. CosMx enables high-plex transcriptomics acquisition via cyclic in situ hybridization chemistry, in which the target gene-binding domain is attached to multiple independent readout domains [106]. To improve the segmentation of individual cells, segmentation markers such as DAPI and PanCK can be used. CosMx enables researchers to combine single-cell transcriptomic data with each cell’s spatial coordinates, allowing analysis of individual microenvironments and cell-to-cell interactions. In IA, there will be opportunities to explore extensive cell-to-cell interactions between infiltrating macrophages and surrounding cells.

### Visium v2 and Visium HD

10x Genomics Visium v2 and Visium HD provide spatial transcriptomic sequencing using gridded sequencing spots [107]. The original Visium had spots 55 micrometers in diameter that contained attached oligonucleotide barcodes for RNA capture [108]. The Visium HD increased spatial resolution to up to 2 micrometers per spot. While the centers of each spot in Visium were 100 micrometers apart, creating a dead space, Visium HD eliminated this dead space by placing spots or bins directly adjacent to one another [109]. FFPE, FF, and fixed-frozen specimens can be used with both Visium v2 and HD. The platforms accept both human and mouse samples, and the whole-transcriptome panels are available for both species. For visualization, Hematoxylin and Eosin (H&E) staining or immunofluorescence can be performed. The entire slide within the designated read area is processed; no ROI delineation is required. If the slide contains both an aneurysm and adjacent tissues, Visium will yield transcriptomic data from all areas within the fiducial frames.

### Xenium in Situ Analyzer

Xenium is an imaging-based spatial transcriptomics platform. Similar to CosMx, Xenium enables imaging-based subcellular-resolution spatial transcriptomics. Transcript-targeting padlock probes and rolling-circle amplification are the bedrock of the Xenium platform, enabling high sensitivity and specificity [110, 111]. Morphological stains, such as DAPI and CD45, are used to improve cell segmentation. Several benchmark articles on spatial transcriptomics indicate that Xenium is highly effective for RNA detection, with high sensitivity and specificity. The Xenium produced data concordance was also reported to be reliable. This could be an advantage when harmonizing Xenium-generated data with publicly available data. However, there is no option to perform a whole-transcriptome search on Xenium. The 5 K panel offers the broadest coverage. It may be advisable to use the platform after other spatial or single-cell transcriptomics platforms.

### Stereo-seq and V2

Stereo-seq is a sequencing-based spatial transcriptomics platform by STOmics. Using DNA nanoball-patterned chips, Stereo-seq reduced the spot size to 0.22 micrometers, achieving a high number of spots per unit area and a large maximum area [22]. The reliable capture sensitivity and specificity were demonstrated by its successful use in studying the mouse embryogenesis. Stereo-seq v2 improved molecular diffusion and expanded the platform to FFPE tissues, as revealed in late 2025 [112]. The Stereo-seq portfolio can accommodate larger whole-sample sizes and enables mounting of a large IA sample on a single slide: 2 cm x 3 cm standard chips are available. This could be especially useful when studying giant IAs with sizes of 25 mm or greater [113]. Nuclear staining and H&E staining are available for visualizing tissue architecture.

## Future Outlook and Conclusion

**Fig. 4:**
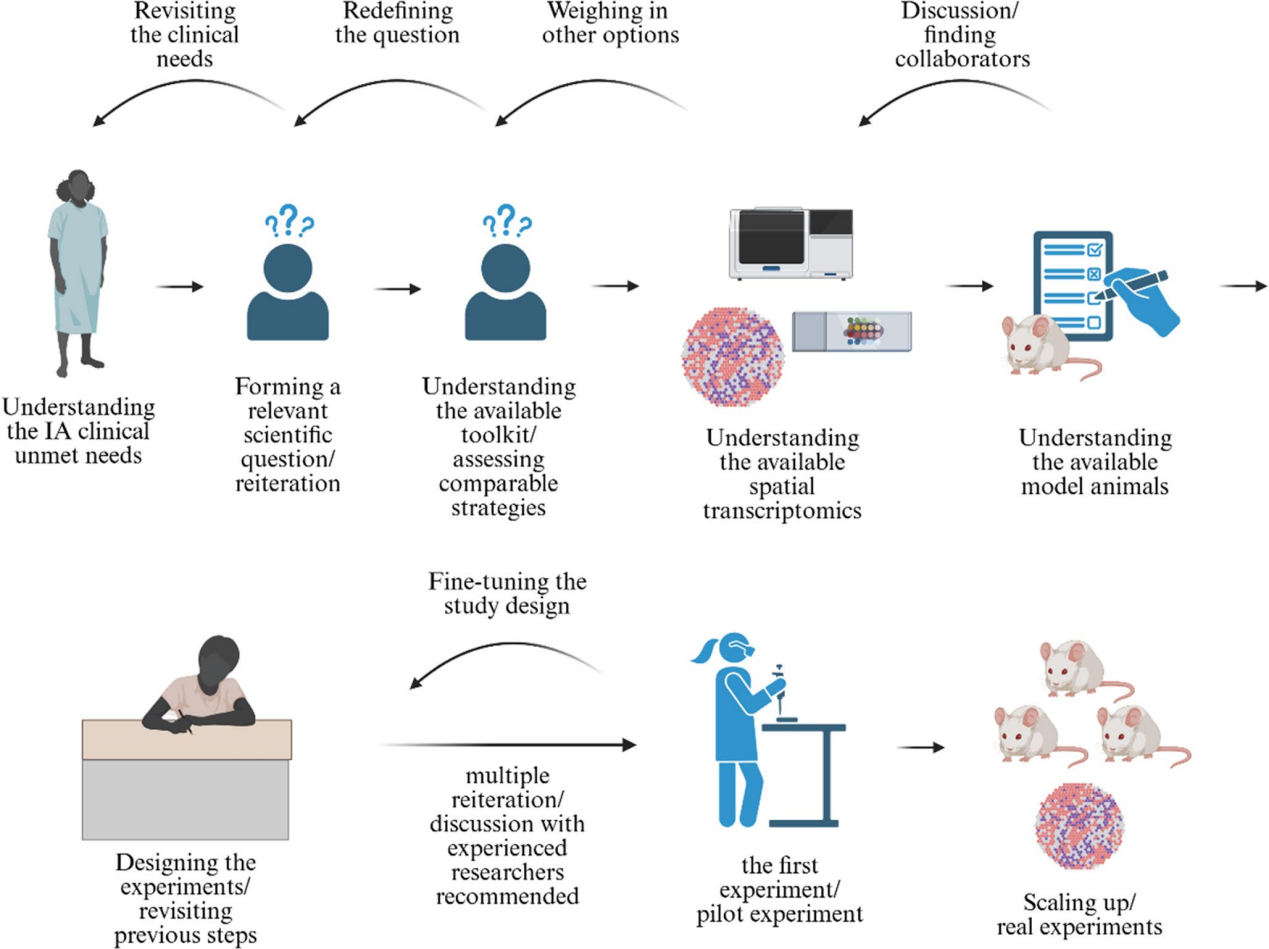
The example logistics for animal model-based spatial transcriptomics are illustrated. While spatial transcriptomics is a powerful toolkit for analyzing potential immune cell contributions to IA, appropriate preparation is recommended to fully leverage it. The authors recommend revisiting previous steps multiple times or conducting multiple iterations during the trial-and-error phase to achieve a reproducible study design. Understanding the limitations of each platform and available animal model is also highly recommended

With the development of spatial transcriptomics technology, the immunological understanding of diseases with unmet clinical needs is evolving rapidly [24]. In IA, infiltrating macrophages and their roles in IA formation, growth, and rupture can be studied using available spatial transcriptomics platforms [114]. However, selecting spatial transcriptomics requires an in-depth evaluation of the scientific questions and an understanding of the technology’s limitations [108, 110, 111] (Fig. 4). In some instances, well-established technologies may be better suited to answering questions than the latest spatial transcriptomics methods [115].

Moreover, scientific questions will be more effective when they acknowledge the heterogeneity of the IA, including patient age, sex, risk factors, and other pertinent information, such as epigenetic factors [2, 116–119]. In particular, multi-omics approaches might hold the key to discoveries with potential therapeutic implications [118, 119]. Unanswered questions with potential clinical significance can be pursued using spatial transcriptomics, including those related to sexual dimorphism and the prediction of high-risk IAs. Although outside the scope of this article, understanding non-saccular IAs could provide critical insights as well.

Immune cell sexual dimorphism is increasingly recognized as complex and nuanced. While distinct from the sexual dimorphism question, the escalation of aneurysmal SAHs in postmenopausal women also needs a close investigation [2, 69]. Taking account of sexual dimorphism in IA appropriately will lead to meaningful insights for developing therapies for women and post-menopausal women, who often experience the heaviest disease-associated burdens [2]. An in-depth understanding of mechanotransduction in IA will also help researchers elucidate how infiltrating immune cells function at arterial bifurcations and potentially inform understanding of high-risk IAs [38]. Some questions can be effectively addressed using animal models despite their limitations [69, 93–95]. Promising results from animal studies can lead to more coordinated efforts toward patient-sample-driven investigations. Well-defined questions will lead to the most appropriate use of IA animal models and spatial transcriptomics, and enable insightful translational research with important therapeutic implications [23, 120, 121].

## Data Availability

No datasets were generated or analysed during the current study.
